# My plea for academic decency remains unchanged: a response to Lior Pachter

**DOI:** 10.1007/s00359-025-01764-3

**Published:** 2025-10-08

**Authors:** Eric J. Warrant

**Affiliations:** Lund Vision Group, Department of Biology, University of Lund, Sölvegatan 35, 22362 Lund, Sweden

**Keywords:** Lior Pachter, Laura Luebbert, Mandyam Srinivasan, Honeybee odometer controversy, Scientific misconduct

## Abstract

Lior Pachter’s response to my Editorial (Warrant in J Comp Physiol A 10.1007/s00359-025-01745-6, 2025)—which details the recent public attacks on the integrity of Australian neuroethologist Mandyam Srinivasan—does little more than accuse me of writing a long series of ad hominem attacks against him and Laura Luebbert. I find this accusation very difficult to reconcile with what I actually wrote, and I leave it to the readers of my Editorial to decide for themselves whether I am guilty of this or not. Regardless of this, Pachter fails to address, or refute, the reason I wrote my Editorial in the first place—namely, to decry the manner in which he and Luebbert raised their concerns about Srinivasan’s work. Thus, the conclusion of my Editorial remains unaltered—Luebbert’s and Pachter’s unjust public assassination of Srinivasan’s reputation falls vastly short of the standards of academic decency expected of a respectful scientific discourse between peers.

❦

*When did virality replace due process? Public shaming is now a participatory sport. Viral justice is gamified. {…} Every new fact unlocked is rewarded in likes and retweets. What starts as accountability often becomes performance. What begins as critique becomes content.*

*Miski Omar, the Guardian, July 22nd 2025* 1

In his response (Pachter 2025) to my recent Editorial (Warrant 2025), Lior Pachter claims that my opinion piece is little more than a litany of errors and personal (ad hominem) attacks on himself and Laura Luebbert. This appears to be the main thrust of his objection to my Editorial, although I do confess, I have great difficulty in understanding how he could come to this conclusion. Yes, I do mention his name 29 times, and yes, I do stand guilty of apparently misrepresenting his and Luebbert’s professions as “bioinformaticians” rather than as “computational biologists” (I apologise). And yes, I failed to note that Luebbert had changed institutions (her affiliation is listed as Caltech on the original arXiv preprint (Luebbert and Pachter 2024), so I am not sure why that was a problem). Either Pachter has a much lower threshold for what he considers to be an ad hominem attack than I do (he mentions my name 23 times in his response, but I certainly don’t feel under personal attack), or we have very different definitions of what an ad hominem attack actually is. I guess we must, because he clearly does not consider the language and manner he has used to publicly assail Srinivasan’s work and reputation (and by inference Srinivasan himself)—examples of which I have given in my Editorial—as ad hominem attacks. In contrast, I do.

Pachter claims—correctly—that I do not address the substance of his and Luebbert’s critiques of Srinivasan’s work. But this was not the purpose of my Editorial. Srinivasan and colleagues have already addressed and debunked these critiques (Srinivasan et al. 2024; Stuart 2024; Stuart et al. 2024; Srinivasan and Tautz 2025). I have nothing more to add. In my Editorial I only provided an outline of the critiques (and Srinivasan’s responses) to provide historical context for the main message I wished to convey—which concerned the manner in which the critiques were handled (not the critiques themselves). Of course, Luebbert and Pachter had every right to publish their critiques on a preprint server (even though I still maintain that they should have raised their concerns with Srinivasan prior to this). It was not the preprint itself that stimulated my Editorial, rather it was how Luebbert and Pachter behaved *after* the preprint was published.

Pachter also claims that I failed to mention the two Expressions of Concern published by the *Journal of Experimental Biology*. Here are the original sentences from my Editorial: “When alerted to these problems, Srinivasan willingly provided a clarification of these errors in “expressions of concern” that are now permanently attached to these papers. Srinivasan also acknowledges these errors and takes sole responsibility for them.” Pachter also accuses me of implying that he and Luebbert wrote Andrew Gelman’s post^2^ describing Srinivasan’s data as a “fraud”—in his eyes more evidence of my apparent ad hominem attacks. Again, here is the original sentence from my Editorial : “On X, Pachter describes Srinivasan’s work as “junk”, and some of Luebbert’s and Pachter’s followers go further, referring to Srinivasan’s data as “fake” and a “fraud”^2^.” How Pachter can conclude that I imply anyone other than Gelman wrote this post mystifies me.

Pachter also strangely implies that it was meaningless for me to quote Valda Vinson, the Executive Editor of *Science*, who wrote the following about Srinivasan’s (2000) paper: “*Science* editorial has evaluated the concerns and stands by the published paper, at this time.^3^. According to Pachter: “this means very little as *Science* (the journal), is known not to correct itself, e.g. *Science* took 15 years to retract the paper claiming that a bacterium can grow using arsenic instead of phosphorus (Wolf 2025), a claim that was immediately debunked (Reaves et al. 2012; Erb et al. 2012)”. It is an odd statement to claim that *Science* “is known not to correct itself“—its editors have retracted large numbers of papers^4^, and usually much earlier than 15 years after publication. Even in the case of the recently retracted paper by Wolfe-Simon and colleagues, the reasons for *Science* delaying its retraction are understandable (Thorp 2025), and they certainly do not reveal an unwillingness for self-correction. Thus, what Valda Vinson wrote about Srinivasan’s paper was of course *highly* meaningful.

In Pachter’s attempts to paint my Editorial as little more than a personal attack on himself and Laura Luebbert (I will leave the readers of my Editorial to decide if this assessment is justified), he thoroughly fails to address its entire *raison d’être*, namely the methods he and Luebbert chose to raise their concerns about Srinivasan’s work. As I clearly explain in my Editorial, and which I have no intention of detailing again, Luebbert and Pachter publicly accused Srinivasan of serious premeditated scientific misconduct, accusations they magnified in the press and in social media (where they collectively have around 50,000 followers). This was accompanied by the publication of a damning new section on Srinivasan’s Wikipedia page and a rapid, and viral, public execution of Srinivasan’s reputation. This occurred without any prior attempt by Luebbert and Pachter to air their concerns with Srinivasan himself, and without providing evidence that any of the actual irregularities they uncovered were deliberate premeditated acts of misconduct designed to elevate Srinivasan’s conclusions for personal gain. These irregularities are present in three papers (not ten), with typographical errors present in one further paper. Although these irregularities are sloppy, embarrassing and regrettable, they were not deliberate or malevolent. In my opinion, this highly public and unjust assassination of a fellow scientist’s reputation falls vastly short of the standards of academic decency expected of a respectful scientific discourse between peers—even if that discourse involves disagreement. Thus, the conclusions of my Editorial remain unaltered, and its message requires no adjustment.

## Data Availability

No datasets were generated or analysed during the current study.

## Footnotes

1
https://www.theguardian.com/commentisfree/2025/jul/22/the-story-of-the-coldplay-couple-unfolded-like-a-soap-opera-but-was-the-pile-on-that-followed-a-proportional-response-ntwnfb.

2
https://statmodeling.stat.columbia.edu/2024/11/07/fake-data-on-the-honeybee-waggle-dance-followed-by-the-inevitable-it-is-important-to-note-that-the-conclusions-of-our-studies-remain-firm-and-sound/.

3 Inserted as an update in the online version of Quaglia (2024) on August 8th 2024: https://www.science.org/content/article/buzzkill-accusations-leveled-research-dancing-bees-measure-distances.

4 For many examples see: https://www.science.org/action/doSearch?AllField=Editorial+retraction.
